# Beyond excellence, friendship and respect: olympic education stakeholders’ priorities for values in a changing world

**DOI:** 10.3389/fspor.2026.1823613

**Published:** 2026-06-02

**Authors:** Yannis Theodorakis, Charalampos Krommidas, Mary Hassandra, Konstantionos Georgiadis

**Affiliations:** 1 Department of Physical Education and Sport Science, School of Physical Education, Sport & Dietetics, University of Thessaly, Trikala, Greece; 2 Department of Sports Organization and Management, University of Peloponnese, Sparta, Greece

**Keywords:** attitudes, Olympic education, Olympic movement, Olympic values, sport governance, sports policy

## Abstract

The emergence of new governance priorities in sport has become a central concern for the Olympic Movement, whose activities are fundamentally grounded in the Olympic values. Traditionally, the Olympic Movement has emphasized three core values—excellence, friendship, and respect. However, contemporary global sports governance and policy challenges call for a critical re-evaluation and possible expansion of this value framework. In response to evolving societal needs, the present study examines the attitudes of Olympic education stakeholders toward a broader set of Olympic values. These include priorities that reflect current global and educational concerns, such as peace, social inclusion, environmental sustainability, and gender equality. By exploring these perspectives, the study seeks to assess whether and how the Olympic value system can adapt to better address the demands of a rapidly changing world. Two-hundred and eighty-six participants (*N* = 286), aged 18 to 79 years and drawn from international summits and seminars, completed attitude scales assessing the perceived importance of seven Olympic values: excellence, friendship, respect, peace, social inclusion, gender equality, and environmental protection. The results demonstrated consistently high ratings across all values and strong positive intercorrelations among them. Peace emerged as the highest-ranked Olympic value, followed closely by respect and friendship. Multiple linear regression analyses with bootstrap estimation indicated that the traditional core values were strongly interconnected with the proposed contemporary values. Gender-based analyses revealed that all Olympic values were rated higher by females than by males. In contrast, no significant differences were observed with respect to participants’ age or educational level. Based on these findings, the study argues for the enrichment and reconsideration of the Olympic value framework through the formal integration of contemporary values into Olympic education and governance agendas, adapted to the challenges posed by the difficult global circumstances facing our planet. By documenting how Olympic education stakeholders prioritise values in a changing global context, the study offers empirical insight into emerging educational and policy directions for Olympic values education frameworks, including OVEP and Olympism 365.

## Introduction

There is a growing body of scholarship examining how sport organizations respond to contemporary challenges associated with diversity, inclusion, and equitable governance (1). Within the broader debate on the evolution of sport governance, increasing attention has been directed toward the effectiveness of governance reforms in addressing structural inequalities, representation deficits, and exclusionary institutional practices. In this context, sport governance is expected not only to ensure transparency, accountability, and organizational effectiveness, but also to promote equity, diversity, and inclusive participation through policies aimed at reducing underrepresentation in decision-making processes. Effective governance frameworks should therefore foster diversity in leadership, gender equality, accessibility, and social participation, reinforcing sport's potential role as a mechanism for social cohesion and democratic inclusion (2).

However, critical scholarship suggests that governance reforms in sport often struggle to produce substantive structural change. Resistance to gender balance within sport governance structures may partially derive from the sport capital and habitus of board members, whose institutional authority can normalize exclusionary judgments and reproduce male-dominated power relations within decision-making environments (3). Such findings raise broader questions regarding whether contemporary governance reforms genuinely challenge entrenched inequalities or merely reproduce them through updated governance discourses and managerial practices.

This tension is particularly evident in the case of the International Olympic Committee (IOC). Although the IOC has achieved important symbolic milestones, including athlete gender parity, its governance approach has been characterized as a form of “nested newness,” whereby equality-oriented reforms are incorporated into pre-existing institutional arrangements without generating substantial transformation. Existing analyses highlight a persistent gap between formal policy commitments and meaningful institutional change. More specifically, the IOC's reliance on quantitative targets and soft governance mechanisms appears insufficient to disrupt deeply embedded masculine norms, resulting in limited progress in leadership representation, coaching opportunities, and the equitable allocation of resources. Consequently, participation parity may function more as symbolic co-optation than as evidence of genuine structural reform. While international sport governing bodies increasingly frame gender equality as a universal normative value, their policies often remain aspirational in the absence of systemic institutional restructuring and enforceable accountability mechanisms (4).

At the same time, governance processes in sport cannot be detached from broader political and socio-economic environments. Women's representation in sport governance, for example, has been shown to correlate closely with state politics and national institutional cultures, suggesting that spillover effects from the political environment significantly shape governance practices within sport organizations (5). This relationship highlights the continuing importance of the state and public policy in shaping governance agendas, despite broader shifts toward network-based, multi-level, and hybrid governance models in sport.

Furthermore, contemporary sport governance increasingly incorporates objectives related to sustainability, social inclusion, urban legacy, and environmental responsibility, particularly in the context of mega-sport events. Nevertheless, important questions remain regarding the long-term effectiveness and sustainability of such governance models. Research has questioned whether ambitious governance objectives can realistically be achieved within restrictive timelines, fragmented institutional structures, and highly complex political environments. Although mega-events such as the Olympic Games may operate as catalysts for investment, urban regeneration, and policy coordination, their long-term territorial and social outcomes depend heavily on sustained political commitment and inclusive governance practices beyond the event itself.

In this regard, Gignon (6) argues that the Paris 2024 Olympic Games represented both an opportunity and a critical test for public governance in France, particularly concerning equitable territorial development in north-east Paris. The success of this governance model depended not only on infrastructure delivery and economic investment, but also on the integration of Olympic projects into broader urban development strategies, the transparency and inclusiveness of governance processes, and the capacity of public authorities to mitigate rather than intensify existing socio-spatial inequalities. These debates ultimately reflect broader concerns within contemporary sport governance research regarding the persistence of structural inequalities, the effectiveness of governance reforms, and the possibility of developing sustainable, inclusive, and evidence-based governance models capable of addressing emerging global challenges in sport.

These broader debates surrounding governance, inclusion, accountability, and institutional reform in sport are closely connected to the ideological foundations and normative principles promoted by the Olympic Movement. Indeed, governance frameworks within international sport organizations are not developed in isolation from the values and philosophical assumptions that underpin Olympism. Rather, the governance priorities of institutions such as the IOC are shaped by broader narratives concerning ethics, education, social responsibility, human rights, and universal inclusion. Consequently, examining the relationship between Olympic values and governance practices becomes essential for understanding how normative ideals are translated into policy agendas, institutional mechanisms, and educational strategies within contemporary sport systems.

Values have long constituted a central concept not only in sociology, but also in psychology, anthropology, and related disciplines. They are employed to characterize cultural groups, societies, and individuals, to examine social change over time, and to explain the motivational foundations underlying attitudes and behavior. In conceptualizing values, individuals typically reflect upon what they consider important in their lives. Each person possesses a wide range of values—such as achievement, security, and benevolence—which differ in their relative significance (7). From an axiological perspective—the branch of philosophy concerned with the nature, hierarchy, and grounding of values—values are not merely personal preferences but socially embedded, motivationally potent orientations that guide judgment, action, and institutional practice. Schwartz's (7) cross-cultural theory identifies ten universal value types organized around two bipolar dimensions (self-enhancement vs. self-transcendence; openness to change vs. conservation), offering a robust theoretical lens through which the prioritization of Olympic values can be interpreted. It is equally important to distinguish conceptually between values and public policy, two constructs that are closely related but analytically distinct. Values, in the Schwartzian sense, are motivational goals that guide individual and collective behavior; they operate as normative orientations and evaluative standards. Public policy, by contrast, refers to formal institutional decisions, regulations, and resource allocations through which states and sport governing bodies translate normative goals into binding or operative frameworks. Olympic values therefore do not automatically become policy: they require institutional mediation, political prioritization, stakeholder negotiation, and accountability mechanisms before they acquire governing force. This study is concerned primarily with the normative layer—stakeholder value priorities—while recognizing that the pathway from values to policy is contested, contingent, and shaped by power relations within sport governance systems. Based on the above, the Olympic Movement and the institution of the Olympic Games have historically cultivated their own distinct system of values. From antiquity to the present day, these values have been continuously redefined and adapted in accordance with evolving social needs and contemporary societal demands.

According to the Olympic Charter, Olympism is presented as a comprehensive philosophy of life that seeks to integrate sport with culture and education, promoting ideals such as the joy of effort, the educational value of good example, social responsibility, and respect for internationally recognized human rights and universal ethical principles (8). While this normative framework positions Olympism as a morally grounded and socially transformative project, its practical implementation remains contested. Olympic values are not simply moral ideals; they also function as educational priorities that shape curricula, pedagogies, and institutional agendas. The values that are prioritised influence what is taught, assessed, funded, and legitimised within Olympic education and related policy frameworks. In this sense, Olympic values are not merely taught; they are also governed, interpreted, and strategically mobilised across institutions and contexts. More broadly, the fundamental values of the Olympic Movement are frequently invoked to legitimise sport organisation at national and international levels, shape the public image of mega-sporting events, and inform the overarching philosophy of sport governance.

However, the extent to which such values will affect policy, practice, and lived sporting experiences, particularly in contexts marked by commercialisation, political interests, and social inequality, has been widely questioned. At the core of Olympism are the values of excellence, friendship, and respect, promoted not only as defining characteristics of elite sport but also as universal principles applicable to everyday life. Nevertheless, tensions persist between these aspirational ideals and the realities of contemporary sport, raising critical concerns about value instrumentalisation, symbolic compliance, and the gap between Olympic rhetoric, educational claims, and practice. In educational contexts, this gap raises questions about whether Olympic values are taught as critical ethical commitments or as symbolic narratives that serve institutional legitimacy.

The value of excellence emphasizes the continuous pursuit of personal improvement. Athletes are encouraged to strive for their best possible performance, setting an example that extends beyond sport and inspires individuals to develop their capabilities in all areas of life. Friendship reflects the unifying power of sport, fostering mutual understanding among people of different ages, cultures, and backgrounds. Through participation in sport and international sporting events, communities and nations are brought closer together, strengthening cooperation not only in sport but also across political, economic, and social domains. Respect underscores the importance of integrity, promoting regard for oneself, one's opponents, the rules, the environment, and society as a whole (8–12). Collectively, these foundational principles anchor the Olympic Movement in its broader mission of contributing to society (13). However, some scholars argue that additional Olympic values should be recognized as fundamental in order to provide a broader and more inclusive perspective—one that more accurately represents both the Olympic and Paralympic Games (14–16).

As issues related to sport governance continue to evolve, new challenges are emerging, highlighting the growing need to focus on governance models that prioritize equality, integrity, transparency, inclusion, and environmental responsibility—values that reflect the broader priorities of modern societies. In this context, the Olympic Movement is likewise called upon to redefine its value system in accordance with these principles. In alignment with these values, the International Olympic Committee (IOC) introduced the Olympism 365: Sport for a Better World—Everyday, Everywhere initiative. This strategic framework links sport to global development priorities, including good health and well-being, quality education, gender equality, reduced inequalities, sustainable cities and communities, responsible consumption and production, climate action, and peace, justice, and strong institutions. Collectively, these efforts reflect the IOC's commitment to extending Olympism beyond elite competition and embedding its philosophy within broader social action. In doing so, the IOC seeks to strengthen the role of sport as a catalyst for achieving the United Nations’ Sustainable Development Goals (17, 18). SDP is widely promoted as a mechanism for achieving social outcomes beyond sport, yet researchers have emphasised that such claims are shaped by power relations, funding logics, and institutional legitimation processes (19, 20).

Sport for Development and Peace (SDP) provides an additional lens through which Olympism is translated into educational and policy practice. SDP refers to the intentional use of sport and physical activity to pursue social outcomes beyond competition, including education and life skills, health and wellbeing, social inclusion, and peacebuilding. At the same time, the SDP field has been critiqued for the ways in which claims about sport's social impact are shaped by power relations, funding logics, and institutional legitimation processes rather than by straightforward educational effectiveness (19, 20). This debate has expanded alongside the growth of multisectoral partnerships involving non-governmental organisations, governments, donors, and sport federations, all of which influence what ‘counts’ as desirable outcomes and how values are framed for learners and communities (21).

These tensions are also visible in participation and equity agendas. Structural barriers to sport participation intersect with broader population trends, including steep declines in physical activity across adolescence and early adulthood and persistent gender gaps at the global level (22, 23). Participation gaps are particularly pronounced among people with disabilities (24), illustrating how inequalities in opportunity and access can undermine the universalist claims often attached to Olympic values. From an educational perspective, such patterns reinforce the need for values education to address not only individual attitudes but also the social conditions that shape who is able to participate, belong, and benefit from sport.

Environmental sustainability further illustrates the contested nature of Olympic values in contemporary sport policy and governance. Critical scholarship has long questioned whether mega-events can be reconciled with ecological responsibility, highlighting how environmental commitments may operate as symbolic compliance rather than structural change (25). While the IOC frames sustainability as a strategic priority across environmental and social responsibility agendas (9), researchers argue that meaningful progress depends on embedding sustainability within governance systems and measurable targets rather than relying on aspirational commitments (26). In SDP and Olympic contexts alike, the educational promise of sustainability therefore remains inseparable from the political economy of events, resource use, and accountability.

Finally, peacebuilding remains one of the most aspirational and debated claims associated with Olympism. Although sport and mega-events are frequently promoted as vehicles for peace and mutual understanding, research emphasises that peacebuilding through sport is not automatic but contingent on context, governance, and sustained institutional engagement (19, 20). The Olympic Truce (ekecheiria) illustrates both the potential and the limits of this narrative: it retains symbolic and normative significance through repeated endorsement in global policy arenas, yet its capacity to generate sustained peace remains contested (27, 28). For Olympic values education, this means that ‘peace’ cannot be approached only as celebratory rhetoric; it requires pedagogical work that engages learners with conflict, inequality, and the practical dilemmas of translating values into action.

There are several noteworthy examples from recent Olympic history, that demonstrate the adaptation and practical promotion of Olympic values by the International Olympic Committee. For instance, the 2024 Summer Olympics marked the first Olympic Games in history to achieve full gender parity, with equal numbers of male and female athletes participating. In the same context, the introduction of mixed-gender events, such as the mixed 4 × 400 relay and the Marathon Race Walk Mixed Relay, reflected the IOC's commitment to gender equality and inclusiveness in sport.

With regard to climate change and environmental sustainability initiatives, the 2020 Summer Olympics introduced medals produced from recycled electronic waste, while recycled plastics were also used in several operational aspects of the Games. Furthermore, the 2024 Summer Olympics placed particular emphasis on sustainable development through the use of 100% renewable electricity for Olympic venues. Approximately 84% of the venues were existing or temporary facilities, thereby limiting the environmental impact associated with new construction. In addition, the Games promoted sustainable transportation through fleets of electric, hydrogen, hybrid, and autonomous vehicles, while also investing in cycling infrastructure, including dedicated bicycle lanes and secure bicycle parking facilities.

In relation to inclusion and the acceptance of diversity, a particularly significant initiative was the establishment of the Refugee Olympic Team at the 2016 Summer Olympics, where ten refugee athletes competed under the Olympic flag. This initiative symbolized the Olympic Movement's commitment to solidarity, human dignity, and the universal right to participate in sport regardless of nationality or displacement.

Based on the above discussion, it appears that the promotion of the three Olympic values, excellence, friendship, and respect, does not fully capture the breadth and dynamic nature of Olympic values. It is widely acknowledged, both in academic literature and within Olympic philosophy, that issues such as gender equality, social inclusion, environmental protection, and peace are of particular importance and are frequently articulated as core Olympic values. Consequently, a reconceptualization of the topic, alongside a contemporary and critical examination of Olympic values, would be both timely and beneficial for the Olympic Movement. These issues feature prominently among the priorities of the Olympic Agenda 2020 + 5 (29).

Gender equality in sport is fundamentally about ensuring that all individuals, regardless of gender, have equal opportunities to participate, compete, and lead. Although global participation by women and girls has increased, substantial disparities persist. Expanding access to sport for women and girls yields wide-ranging social benefits, particularly in relation to public health outcomes (30). Gender equality has long been central to the IOC's mission, formally embedded in the Olympic Charter and reinforced through initiatives aimed at balanced participation and leadership representation. Collaboration with international sport federations and National Olympic Committees has resulted in targeted action plans to advance equality across all levels of sport governance. Significant progress has been achieved in recent Olympic Games, culminating in full gender parity at the Paris 2024 Olympic Games, including parity targets and reporting on women's participation and representation (10). Research emphasizes that targeted policies, educational strategies, and Olympic education initiatives can promote human rights awareness, challenge gender stereotypes, and foster equitable opportunities in sport (31).

Social inclusion and human rights are firmly embedded within the IOC framework, grounded in the Olympic Charter's recognition of sport as a fundamental human right and its commitment to non-discrimination. The Olympic Movement stands against discrimination of any kind, including race, colour, gender, sexual orientation, social, language, religion, political belief or social background. Despite increasing accessibility, substantial barriers to sport participation persist for certain groups, including ethnic minorities, individuals from low-income backgrounds, people with disabilities, older adults, and sexual minorities. Obstacles such as unsafe environments, inadequate transportation, time constraints, and social exclusion continue to restrict participation (32). Structural barriers to participation intersect with broader population trends, including steep declines in physical activity across adolescence and early adulthood and persistent gender gaps at the global level (22, 23). Participation gaps are particularly pronounced among people with disabilities (24).

Environmental sustainability in sport has become a contested global issue, particularly in relation to the environmental costs of mega-sporting events. As the scale and frequency of such events increase, their ecological impacts have drawn heightened scrutiny. While education for sustainable development is widely promoted as a means of fostering sustainability awareness and action (33), sport's role remains ambivalent, functioning both as a contributor to environmental degradation and as a claimed platform for sustainability promotion. These debates intersect with sustainability commitments framed through IOC environmental and social responsibility agendas (9). Researchers argue that environmental progress depends on embedding sustainability within governance systems and measurable targets rather than relying on aspirational commitments (26).

Despite this growing scholarly and institutional attention, a critical gap remains: there is limited empirical evidence on how Olympic education stakeholders—those who translate Olympism into practice across educational, institutional, and policy settings—actually prioritize values in a rapidly changing world. Existing research has mapped the formal value frameworks of institutions such as the IOC (12) and assessed participation patterns (22), but few studies have gathered direct stakeholder attitudinal data on an expanded set of values that includes contemporary governance priorities such as peace, gender equality, social inclusion, and environmental protection. This study addresses that gap by providing the first large-scale, multi-national empirical assessment of stakeholder value priorities across both traditional and contemporary Olympic values, using an internationally representative sample drawn from IOA educational activities. Against this background, the present study addresses the following research questions:

**RQ1.** Which Olympic values do stakeholders in Olympic education prioritise as most relevant in today's world and within contemporary sport governance concerns, practices, and priorities?
**RQ2.** How do traditional Olympic values relate to contemporary societal values—such as peace, inclusion, gender equality, and environmental sustainability—in stakeholders’ perceptions and within sport governance?
**RQ3.** What implications do these priorities have for the future direction of Olympic values education frameworks and pedagogical practices?

## Method

### Participants

A total of 286 participants who attended summits and summer schools at the International Olympic Academy (IOA) during the summer period of 2025 voluntarily participated in the present study. Participants ranged in age from 18 to 79 years (Mage = 33.54, SD = 13.24) and represented 95 countries worldwide. Of the total sample, 129 participants identified as male and 141 as female, while 16 participants did not report their gender. With regard to educational attainment, 19 participants were high school graduates, 120 held a university degree, 95 held a master's degree, and 29 held a doctoral degree, while 23 participants did not report their educational background.

The International Olympic Academy serves as the global center for education in and through Olympism. It organizes its own educational sessions and programs and hosts events for organizations that promote Olympic values at its premises in Ancient Olympia, Greece. Each year, the IOA hosts a wide range of symposia, summits, congresses, and seminars in collaboration with institutions such as the International Scholars’ Symposium on Sports, Society and Culture, Harvard University's Hellenic Studies Program, the International Olympic Truce Centre, the British Council, the Hellenic Cultural and Educational Club for UNESCO, as well as educational institutions, National Olympic Academies, and National Olympic Committees from around the world. In addition, events are organized in cooperation with governmental and non-governmental organizations, researchers, and university-based scholars (34).

More specifically, data were collected during the following IOA educational activities held in the summer of 2025: the 18th International Session for National Olympic Academies and National Olympic Committees Delegates; the 15th International Session for Educators of Higher Institutes of Physical Education; the 65th International Session for Young Olympic Ambassadors; and the 32nd International Seminar on Olympic Studies for Postgraduate Students. Consequently, all participants were familiar with the concepts and discourse surrounding Olympic values. Participants constitute a transnational Olympic education community, including educators, youth ambassadors, NOA/NOC delegates, and postgraduate students. These groups can be understood as Olympic education practitioners and trainees, and as values-education intermediaries who often support the translation of Olympism into educational practice across different national contexts. As such, they may also function as curriculum and policy carriers within institutions connected to Olympic education and values-based sport initiatives. As participants in IOA educational programs (including NOA/NOC delegates, educators, young Olympic ambassadors, and postgraduate students), they constitute a stakeholder community that often functions as intermediaries translating Olympism into educational, institutional, and policy-related contexts.

The IOA's values education approach, as reflected in the Olympic Values Education Programme (OVEP) and the IOC's foundational OVEP toolkit (18), combines elements of value clarification (encouraging learners to reflect on and articulate their own values), value analysis (examining evidence and reasoning about value claims), and experiential learning through sport activities. The curriculum promotes the three core values—excellence, friendship, and respect—through thematic modules that include sport activities, discussion sessions, and reflective exercises. Moral reasoning and Socratic dialogue are used to connect these values to real-world ethical dilemmas, though direct instruction elements are also present, particularly in structured presentations on Olympism and the Olympic Charter. Hidden curriculum effects may also be at play insofar as the normative environment of Ancient Olympia itself contextualizes participants’ engagement with Olympic ideals (35). Crucially, as noted below, participants in the present study were not exposed to targeted values instruction during their IOA stay, which reduces concerns about demand effects in the attitudinal data. All participants, during their stay at the IOA, did not take part in any specific educational seminar aimed at analysing or providing direction, critical reflection, and guidance on Olympic values. Rather, they participated only in broader discussions covering a variety of topics related to the Olympic Games and Olympism, including political, economic, institutional, and educational issues.

Participants were asked to complete scales assessing their attitudes toward a range of Olympic values. Prior to the assessment of each value, a brief description outlining its definition, as derived from official documents of the International Olympic Committee (IOC) and the United Nations (UN) (see Table 1), was provided to ensure conceptual consistency across the study. The research instrument was completed in either English or French, the two official languages of the International Olympic Academy.

**Table 1:** Definitions of the Olympic values examined.

Value	definition
1	“The value of **Excellence** emphasizes that athletes should constantly strive to improve themselves, setting an example for all people to follow in their daily lives — to always aim to become better. along with relevant examples of such behaviors” (11).
2	The value of **Friendship** emphasizes that through sport, people of all ages come closer together. Nations also build stronger connections and cooperate — not only in sports venues and major sporting events but also in everyday life, at political, economic, and social levels (11).
3	The value of **Respect** reminds us that through sports, people learn to respect themselves, their opponents, the rules, the environment, and society. And of course, everyone should carry this respect into their daily lives as well (11).
4	The Olympic value of **inclusion** through sports is about using sport as a universal language to bring people together, no matter their background, nationality, gender, ability, culture, or beliefs (36).
5	The value of **environmental protection** through sports is about recognizing the responsibility of athletes, organizations, and fans to protect the natural world that makes sport possible (9).
6	The value of gender equality through sports is about ensuring that everyone, regardless of gender, has equal opportunities to participate, compete, lead, and be celebrated in the world of sport (10, 36)
7	The Olympic value of Peace is about using sport as a tool to promote harmony, understanding, and goodwill among nations and people (37, 38).

Participants received instructions on how to complete the survey and were assured that there were no right or wrong answers. Completing the questionnaires took approximately 7 min.

To assess attitudes toward Olympic values, the scales were constructed in accordance with Ajzen's recommendations (39) and relevant empirical studies in the field (40). For each value participants evaluated a common stem. For example: “*If the Olympic Movement gives priority to promoting the value of excellence through sport, it is…”*. Responses were recorded using six 7-point bipolar adjective scales, including *useless–useful*, *unpleasant–pleasant*, *bad–good*, *sad–happy*, *ugly–pretty*, and *foolish–wise*.

Finally, participants were asked to evaluate the relative importance of each value by responding on a 10-point scale ranging from *least important* to *most important*. This was introduced with the question: “*Which value or values do you consider the most important and most relevant in today's world?”* Using this procedure, attitudes toward the following values were assessed: excellence, friendship, respect, peace, inclusion, gender equality, and environmental protection.

#### Statistical analyses

Statistical analyses were performed using IBM SPSS Statistics software version 29. The level of significance was set at *p* < .05. Initially, separate Exploratory Factor Analyses (EFA) were conducted to assess the factorial validity of the Olympic values scales. The criteria used for the EFA were Principal Component Analysis (PCA) with direct oblimin rotation (the factors were correlated), the Kaiser-Meyer-Olkin (KMO) measure of sampling adequacy (>.70) and Bartlett's Test of Sphericity, with eigenvalues greater than one, and a factor loading greater than.30 (41, 42). Descriptive statistics (mean, standard deviation) and reliability analysis with Cronbach's alpha were initially calculated. Next, the normal distribution of the data was checked using the Kolmogorov–Smirnov test. None of the examined variables had a normal distribution (*p* < .05). Thus, non-parametric tests were applied. Spearman's rho correlation analysis was used to investigate the relationships between the examined variables. Multiple linear regression analyses with bootstrap estimation were applied having as independent variables the Olympic values of excellence, friendship, and respect and as dependent variables the Olympic values of peace, inclusion, environment, and equality. Although the distributions deviated from normality, regression coefficients were estimated using bias-corrected bootstrapped confidence intervals, which reduces reliance on normality assumptions. When the data are not normally distributed, bootstrapping is a statistical technique that involves repeating the observed data at least 1000 times in order to precisely determine the Confidence Interval (CI) [e.g. (43, 44),]. Then, separate Mann–Whitney U tests were applied to check for possible differences in the Olympic values due to gender (male, female), and age group (based on their median age of 29 years, volunteers were divided into two age groups: Group 1 = Participants’ age equal to or less than 29 years and Group 2 = Participants’ age equal to or greater than 30 years). Finally, separate Kruskal–Wallis H tests were performed to check for possible differences in the Olympic values due to different educational level (high school, university degree, master degree, and doctorate).

## Results

### Exploratory factor analyses (EFA)

EFA supported the factorial validity of the Olympic values scales. For each scale, two components were extracted based on eigenvalues greater than one: a cognitive factor and an affective-emotional factor. These two factors reflect the cognitive and affective nature of attitudes and are fully consistent with the relevant theory of attitudes and their assessment (45). Table S1 (Supplementary File) presents the full factor loading matrices for each of the seven Olympic values scales. Across all scales, items loaded cleanly onto two factors—a cognitive factor (comprising the rational/evaluative bipolar adjective pairs: useless–useful, bad–good, foolish–wise) and an affective-emotional factor (comprising: unpleasant–pleasant, sad–happy, ugly–pretty). Factor loadings ranged from.70 to.94 on the cognitive factor and from.65 to.91 on the affective-emotional factor across scales, all exceeding the.30 threshold. KMO values ranged from.73 to.80 (all above the.70 criterion), and Bartlett's tests were significant (all *p* < .001), confirming sampling adequacy and factor extractability. The two-factor solution accounted for 72% to 79% of total variance across the seven scales. Content validity was established through grounding each scale item in established attitudinal measurement theory (39, 45) and by deriving value definitions directly from official IOC and UN documents (see Table 1), ensuring conceptual alignment between constructs and measurement items. Construct validity is supported by the convergent and discriminant patterns in the Spearman correlations (Table 2): all values correlate positively and significantly with each other (convergent validity), yet the correlations are not so high as to suggest redundancy (discriminant validity; highest r = .73, between Friendship and Respect). Internal reliability (Cronbach's alpha) was satisfactory for all scales (.79 to.87). External reliability was not formally assessed given the cross-sectional design; however, the scales are adaptations of well-validated attitude instruments with established psychometric track records (40).

**Table 2:** Descriptive statistics, Cronbach's alpha reliability index, Kolmogorov–Smirnov test of normality, and Spearman's correlation analysis.

Variables	*M*	*SD*	*A*	K-S	1	2	3	4	5	6	7
1. Excellence	5.94	.99	.79	.14***	-						
2. Friendship	6.28	.91	.81	.21***	.60**	-					
3. Respect	6.29	.94	.81	.22***	.54**	.73**	-				
4. Inclusion	5.20	.84	.83	.23***	.57**	.67**	.72**	-			
5. Environment	6.11	1.01	.85	.19***	.53**	.60**	.62**	.62**	-		
6. Equality	6.15	1.07	.87	.22***	.45**	.58**	.58**	.62**	.63**	-	
7. Peace	6.42	.95	.85	.27***	.43**	.61**	.65**	.62**	.60**	.65**	-

*M*, Mean; *SD*, Standard Deviation; *α* , Cronbach's alpha reliability index; K-S, Kolmogorov–Smirnov test of normality.

** *p* < .01.

### Descriptive statistics, reliability index, normality test, and correlation analysis

None of the examined variables had a normal distribution (*p* < .05). Descriptive statistics (mean, standard deviation), Cronbach's alpha reliability index, Kolmogorov–Smirnov test of normality, and Spearman's *rho* correlation analysis are presented in Table 2. More specifically, all variables showed acceptable reliability indices (from.79 to.87). The mean scores were high across all Olympic values. There were also positive and moderate to high correlations between the Olympic values examined.

### Multiple linear regression analyses with bootstrap estimation

Multiple linear regression analyses with bootstrap estimation showed that Friendship (*B* = .34, BC 95% CI = .15 to.60) and Respect (*B* = .41, BC 95% CI = .17 to.64) were significant predictors of participants’ Olympic value of Peace. Friendship predicted 7.62%, while Respect explained 11.56% of the variance in Peace, respectively. Similarly, Friendship (*B* = .22, BC 95% CI = .03 to.44) and Respect (*B* = .41, BC 95% CI = .20 to.60) were significant predictors of participants’ Olympic value of Inclusion. Friendship predicted 4.41%, while Respect explained 15.13% of the variance in Inclusion, respectively. Furthermore, Excellence (*B* = .17, BC 95% CI = .02 to.33) and Respect (*B* = .38, BC 95% CI = .12 to.61) were significant predictors of participants’ Olympic value of Environment. Excellence predicted 2.43%, while Respect explained 6.30% of the variance in Environment, respectively. Finally, Excellence (*B* = .15, BC 95% CI = .01 to.30) and Friendship (*B* = .38, BC 95% CI = .10 to.66) were significant predictors of participants’ Olympic value of Equality. Excellence predicted 1.59%, while Friendship explained 5.06% of the variance in Equality, respectively. All multiple regression analyses with bootstrap estimation are presented in Table 3.

**Table 3:** Multiple linear regression analyses with bootstrap estimation.

DVs	IVs					Bootstrap^a^				
		*R*	*R^2^*	*F*	*B*	Bias	SE	*p*	BC 95% CI	
									LL	UL
Peace	(Constant)	.70	.49	78.09***	1.67	−.01	.47	.002	.78	2.59
	Excellence				−.00	−.00	.05	.979	−.12	.10
	Friendship				.34	.01	.11	.002	.15	.60
	Respect				.41	−.01	.12	.001	.17	.64
Inclusion	(Constant)	.74	.55	99.52***	.79	−.02	.35	.023	.11	1.44
	Excellence				.08	.01	.05	.126	−.01	.19
	Friendship				.22	.00	.11	.052	.03	.44
	Respect				.41	−.01	.10	.001	.20	.60
Environment	(Constant)	.61	.37	48.49***	1.47	−.01	.46	.002	.57	2.37
	Excellence				.17	.00	.08	.026	.02	.33
	Friendship				.20	.00	.15	.184	−.10	.50
	Respect				.38	−.00	.13	.003	.12	.61
Equality	(Constant)	.58	.34	40.91***	1.46	−.04	.48	.003	.52	2.37
	Excellence				.15	.01	.07	.043	.01	.30
	Friendship				.38	.00	.14	.010	.10	.66
	Respect				.24	.00	.14	.099	−.06	.51

IVs , Independent Variables; DVs, Dependent Variables; SE, Standard Error; BC, Bias corrected; CI, Confidence Interval; LL, Lower Limit; UL, Upper Limit.

a Results with bootstrap estimation are based on 1,000 bootstrap samples.

**p < .01; ** *p* < .01; ***p < .001.

### Gender differences in the Olympic values

Separate Mann–Whitney *U* tests revealed significant differences in Excellence (*U* = 6,482.50, *Z* = −2.24, *p* < .05), Friendship (*U* = 6,484.00, *Z* = −2.35, *p* < .05), Respect (*U* = 5,948.00, *Z* = −3.46, *p* < .001), Inclusion (*U* = 6,177.00, *Z* = −2.96, *p* < .01), Environment (*U* = 6,640.00, *Z* = −2.28, *p* < .05), Equality (*U* = 5,864.50, *Z* = −3.62, *p* < .001), and Peace (*U* = 6,241.00, *Z* = −3.04, *p* < .01) between males and females. More specifically, females reported higher scores in all the Olympic values compared with males (Table 4).

**Table 4:** Gender mean ranks and significant differences in the Olympic values.

Variables	Male (Mean Rank)	Female (Mean Rank)
Excellence *	114.64	134.96
Friendship *	114.49	135.50
Respect ***	110.16	140.74
Inclusion **	112.13	138.24
Environment *	115.93	136.42
Equality ***	109.37	141.23
Peace **	112.58	138.49

* *p* < .05, ** *p* < .01, *** *p* < .001.

### Age group differences in the Olympic values

Separate Mann–Whitney *U* tests revealed no significant differences in Excellence (*U* = 6,804.50, *Z* = −1.17, *p* = .243), Friendship (*U* = 6,739.50, *Z* = −1.39, *p* = .164), Respect (*U* = 7,079.50, *Z* = -.91, *p* = .361), Inclusion (*U* = 7,172.00, *Z* = -.61, *p* = .544), Environment (*U* = 6,717.00, *Z* = −1.68, *p* = .09), Equality (*U* = 6,887.00, *Z* = −1.09, *p* = .274), and Peace (*U* = 7,004.50, *Z* = -.96, *p* = .339) between the two age groups (Group 1: equal to or less than 29 years old and Group 2: age equal to or greater than 30 years old; Table 5).

**Table 5:** Mean ranks of age groups in the Olympic values.

Variables	≤ 29 years old (Mean Rank)	≥ 30 years old (Mean Rank)
Excellence	120.67	131.46
Friendship	120.43	133.11
Respect	122.70	130.91
Inclusion	123.31	128.78
Environment	120.28	135.65
Equality	121.61	131.43
Peace	122.20	130.46

### Differences in the Olympic values due to educational level

Separate Kruskal–Wallis *H* tests revealed no significant differences in Excellence [*χ^2^* (3) = 5.010, *p* = .171], Friendship [*χ^2^* (3) = 1.982, *p* = .576], Respect [*χ^2^* (3) = 4.093, *p* = .252], Inclusion [*χ^2^* (3) = 1.522, *p* = .677], Environment [*χ^2^* (3) = .637, *p* = .888], Equality [*χ^2^* (3) = 1.958, *p* = .581], and Peace [*χ^2^* (3) = 2.035, *p* = .565] due to participants’ educational background (high school students, university degree, master's degree, and doctorate; Table 6).

**Table 6:** Mean ranks of educational level in the Olympic values.

Variables	High school (Mean Rank)	University (Mean Rank)	Master (Mean Rank)	Doctorate (Mean Rank)
Excellence	96.47	127.43	116.71	138.07
Friendship	110.33	123.85	117.83	135.61
Respect	104.44	131.10	115.09	122.46
Inclusion	128.36	125.25	114.90	127.20
Environment	122.74	122.07	121.13	132.73
Equality	113.11	127.57	115.57	126.23
Peace	134.42	125.01	114.75	124.38

## Discussion

In the official International Olympic Committee, *The fundamentals of Olympic values education* (2,023), emphasis is placed primarily on the three core Olympic values of excellence, friendship, and respect. Nevertheless, there is a growing need to critically reconsider and expand this framework in response to the complex challenges highlighted within contemporary sport policy and sport governance. Future-oriented Olympic values education should therefore engage more directly with emerging social transformations and the governance issues that modern sport institutions are increasingly called upon to confront.

The present study examined how Olympic education stakeholders prioritise and evaluate both traditional (excellence, friendship, respect) and contemporary (peace, social inclusion, gender equality, environmental protection) Olympic values in a changing global context. Across the seven values, participants reported consistently high ratings, suggesting broad normative endorsement of an expanded Olympic value framework. Importantly, this stakeholder group constitutes a transnational Olympic education community, including educators, youth ambassadors, NOA/NOC delegates, and postgraduate students, who often function as intermediaries translating Olympism into educational and institutional settings. As such, their value priorities can be understood as potential indicators of emerging directions in Olympic values education and of how particular principles may diffuse across curricula, programs, and policy discourse in different national contexts. Notably, peace emerged as the highest-ranked value, followed closely by respect and friendship, indicating that stakeholders interpret Olympism increasingly through priorities associated with social cohesion, ethical responsibility, and global coexistence. The strong positive intercorrelations among all values, together with regression findings showing that the traditional core values were strongly interconnected with and predictive of the contemporary values, suggest that stakeholders tend to perceive these principles as complementary and mutually reinforcing rather than competing for primacy.

These empirical priorities are not merely attitudinal: they carry concrete governance implications that can be traced through recent IOC policy history. The IOC's Gender Equality and Inclusion Progress Report 2021–2024 (IOC, 2024) documents measurable institutional outcomes of value-driven governance reform, including the introduction of gender parity targets for NOC leadership (40% women on all NOC boards by 2025), the creation of the Olympic Refuge Foundation following stakeholder and civil society pressure, and the formal embedding of sustainability criteria into host city contracting through the New Norm reforms initiated after 2017. These examples illustrate how the normative prioritization of values such as gender equality, inclusion, and environmental protection by governance actors has been translated—however imperfectly—into binding institutional requirements and accountability frameworks. The stakeholder priorities identified in this study mirror precisely these governance directions: peace, respect, inclusion, and equality are not merely educational aspirations but normative anchors around which governance reform in the Olympic Movement has been organized. Conversely, the persistence of institutional gaps—such as the slow progress on women in coaching and leadership despite formal policy commitments (4)—underscores why stakeholder endorsement of values alone is insufficient without enforceable mechanisms. This study therefore situates its findings at the interface between normative value priorities and governance accountability: understanding what stakeholders value a necessary but not sufficient condition for governance change is. These findings have direct implications for Olympic values education and its wider policy orientation. Although Olympism is positioned as an educational philosophy, Olympic education is often implemented in short-term, event-centered, and fragmented ways, which can reinforce a gap between values discourse and pedagogical practice. Within this context, the prioritisation of contemporary values such as peace, inclusion, equality, and environmental protection underscores the importance of strengthening the educational coherence and societal relevance of Olympic education, including greater clarity about what is taught, how it is taught, and what kinds of civic and ethical outcomes it seeks to promote. At the same time, because values remain contested and can be strategically mobilised, these results invite critical reflection on whether Olympic values are enacted as substantive ethical commitments in educational settings or function primarily as symbolic narratives that support institutional legitimacy.

The demographic analyses further reinforce the socially situated nature of values education. Females rated all Olympic values higher than males, whereas no significant differences were observed across age or educational level. This pattern suggests that engagement with Olympic values may be shaped by gendered experiences and socialisation, with potential relevance for the design and delivery of Olympic education initiatives and for understanding how different groups may connect to values-based pedagogical messages. Taken together, the results support the argument that contemporary Olympic values education frameworks should not only acknowledge an expanded set of values but also attend to how these values are translated into sustained educational practices, institutional strategies, and policy commitments.

The finding that peace emerged as the highest-ranked value among this transnational stakeholder community carries particular sociological significance given the geopolitical context of 2025–2026, characterized by ongoing armed conflicts, rising nationalist tensions, and an erosion of multilateral norms. Sociologically, this result can be interpreted through Giulianotti's (2016) framework of Sport for Development and Peace: stakeholders embedded in an institution explicitly committed to Olympism's peace narrative are likely to reproduce and amplify that institutional discourse. Yet the finding may also reflect a genuine and contingent social response—a demand signal from practitioners on the ground that the Olympic Movement engage more seriously with peacebuilding as a governance priority, not merely a rhetorical one. Critically, as Burleson (27) and the United Nations (28) have noted, the Olympic Truce (ekecheiria) retains symbolic salience but limited operational effectiveness; translating peace as a value into governance mechanisms requires sustained institutional engagement, dedicated funding streams, and cross-sector partnerships that go well beyond the biennial declaration of the Truce. The fact that peace was rated highest by this stakeholder group—above even the traditional core values—may therefore constitute a demand for more substantive institutional investment in peace-oriented programming within Olympic governance frameworks. Sport remains a universal cultural phenomenon with significant potential to influence social norms, educational agendas, and public policy. Within Olympic and broader sport-for-development discourses, sport is frequently positioned as a vehicle for cultivating ethically informed citizenship, strengthening social cohesion, and addressing pressing global challenges. However, the translation of such ambitions into meaningful outcomes is neither automatic nor politically neutral; it depends on how values are defined, prioritised, and operationalised through coherent pedagogical approaches, institutional strategies, and governance commitments. In this sense, stakeholders’ prioritisation of peace, social inclusion, gender equality, and environmental protection is not only descriptive of contemporary moral concerns but also indicative of the directions that Olympic values education may need to take in order to remain socially relevant and educationally credible. Building on this, the following sections discuss how each of these contemporary values aligns with and also challenges, existing Olympic education narratives, and what their formal recognition could imply for future educational frameworks and policy practice.

Values frameworks are never politically neutral: prioritising some values over others can shape whose experiences are recognised, whose participation is supported, and what social issues are treated as “Olympic” or worthy of institutional attention. Stakeholder priorities therefore operate not merely as individual preferences but as signals of educational legitimacy and policy direction, influencing curriculum emphasis, resource allocation, and the ways sport institutions present their social responsibilities to wider publics. From this perspective, the strong endorsement of contemporary values may be interpreted not only as a moral orientation but also as a demand for greater alignment between values-based rhetoric and accountable institutional practice.

From a governance perspective, the strong endorsement of gender equality by female stakeholders in this study aligns with what Knoppers et al. (3) describe as the structural challenge of institutional reform: it is precisely those most affected by inequality who are most normatively invested in its redress, yet their agency within decision-making structures remains constrained by sport capital and masculine habitus. This tension between stakeholder value priorities and institutional governance realities represents one of the central accountability gaps that sport governance reform must address. Promoting gender equality through targeted policies, particularly those aimed at increasing participation among girls and women, remains essential. Such efforts are most effective when pursued alongside initiatives addressing environmental sustainability, social inclusion, and peace, and when supported by accountability mechanisms that move beyond symbolic commitments. Migration contexts further highlight the importance of inclusive sport policy and practice. Limited resources, language barriers, and reduced social and cultural capital continue to constrain participation among refugee and migrant populations. Sport practitioners must recognize the diversity of migrants’ sporting backgrounds, shaped by prior exposure to organised sport structures, geographic proximity, and mobility patterns. Sensitivity to historical and contemporary sociopolitical factors influencing migrants’ experiences of sport participation is therefore essential (46).

Despite ongoing efforts, the Olympic Movement continues to face persistent challenges related to gender equality, social inclusion, environmental sustainability, and human rights. Women and girls remain less physically active than men and are underrepresented in leadership positions and media coverage, particularly when gender intersects with disability or sexual minority status (47, 48). Social exclusion in sport continues to affect ethnic minorities, individuals from low-income backgrounds, people with disabilities, older adults, and sexual minorities due to structural barriers such as limited access, safety concerns, and exclusionary social environments (32).

Olympic Education represents a central mechanism for cultivating Olympic values by linking schools with broader societal issues and highlighting the humanistic, cultural, and ethical dimensions of Olympism. Such programs promote cooperation, respect, inclusion, and critical thinking, while also raising awareness of key contemporary challenges, including gender equality, environmental protection, racism, and social exclusion (35, 49). However, Olympic education initiatives are often fragmented and implemented on a short-term basis around the Olympic Games, which constrains their long-term educational impact and limits opportunities for sustained integration into school curricula. Within this context, Olympic education is closely connected to volunteerism, as both aim to foster civic responsibility and active citizenship. Although Olympic volunteerism offers a wide range of civic engagement opportunities, sustained benefits depend on continued attention to post-event selection and support mechanisms. Accordingly, it has been suggested that event organisers develop structured pathways that introduce and connect volunteers to a broader spectrum of civic engagement opportunities following mega-sport events (50).

While sport and mega-events are often promoted as vehicles for peace and mutual understanding, their potential can only be realised through sustained engagement with contemporary global challenges. Scholars and critics argue that many sport initiatives remain largely symbolic, calling for more substantive and systematic action on inclusion, gender equity, physical inactivity, athlete welfare, climate change, and environmental impact (51). Sport's global reach nonetheless provides a powerful platform for advancing sustainable development across health, education, gender equality, and climate action (52, 53). The challenge for Olympic values education is therefore not only to communicate sustainability as a principle, but to engage learners critically with how environmental priorities are negotiated in practice and how contradictions between sustainability discourse and the ecological costs of sport systems are addressed.

The Olympic Values Education Program (OVEP), centered on Excellence, Respect, and Friendship, offers a strong foundation for youth engagement. However, in light of contemporary challenges and the priorities identified by stakeholders in this study, Olympic values education frameworks may benefit from further development to reflect peace, social inclusion, gender equality, and environmental protection more explicitly and systematically. Three implications are especially important. First, curriculum content may require revision, so that contemporary values are formally integrated alongside the traditional core rather than treated as optional or peripheral themes. Second, a pedagogical shift is needed, moving beyond celebrating values as slogans toward critical values pedagogy that engages learners with ethical dilemmas, power relations, inequality, and the ecological contradictions of mega-events and sport systems. Third, teacher education and educator training should be strengthened, supported by practical modules and resources that enable educators to translate Olympic values into sustained learning experiences across school physical education, youth sport, and community sport settings. In parallel, greater cross-sector collaboration among policymakers, sport organizations, educators, and environmental agencies remains essential to promote physical activity, sustainable mobility, and climate awareness. Aligning sport mega-events with environmental responsibility, such as the climate-positive ambitions of Paris 2024, represents a critical opportunity for the coming decade (54).

Several limitations should be acknowledged. First, the sample consisted of IOA-connected Olympic education stakeholders and should not be interpreted as representative of the general population. Second, uniformly high value ratings may partly reflect social desirability and the normative context of Olympism-oriented educational settings. Third, the cross-sectional design captures priorities at a single time point and cannot address change over time. Finally, prioritising values does not necessarily translate into consistent pedagogical implementation; future research should examine how values are enacted in practice across curricula, teacher education, and community sport programmes, including the institutional conditions that facilitate or constrain sustained values-based education.

In today's complex and rapidly changing world, a persistent gap remains between the ideals of the Olympic Games, the realities of contemporary sport, the ambitions of major event organizers, and the transformative potential of well-designed educational initiatives in Olympic education. While Olympic values promote excellence, respect, and friendship, their consistent and meaningful application in practice is not always guaranteed. For this reason, all stakeholders—sporting institutions, policymakers, educators, and civil society—must renew their commitment to fostering a better world through sport. This effort requires not only the preservation of enduring Olympic values, but also their critical enrichment and reinterpretation in light of present social realities. For example, the value of peace should be more prominently highlighted and systematically embedded within Olympic education programs. By doing so, Olympic education can contribute more effectively to the enrichment and reconsideration of the Olympic value framework through the formal integration of contemporary values, adapted to the pressing global challenges facing our planet

The transition towards a post-governance era in sport, and particularly in mega-events such as the Olympic Games, requires the adoption of innovative, adaptive, and sustainable governance models capable of responding to increasing complexity and uncertainty in the global sport environment. Contemporary societies are placing growing emphasis on issues of equality, inclusion, representation, environmental governance, and sustainability in sport, which now cut across political, economic, institutional, and educational domains with increasing urgency and visibility.

Against this backdrop, a critical re-evaluation and strategic expansion of Olympic values, aligned with contemporary social, cultural, and environmental transformations, could play a pivotal role. Such a process would not only reinforce the normative relevance of the Olympic Movement but also contribute to strengthening the effectiveness, legitimacy, and responsiveness of governance mechanisms in sport. In this sense, the evolution of Olympic values may serve as a key driver for addressing the multifaceted challenges facing contemporary sport systems and for promoting more resilient and socially responsive models of sport governance.

In conclusion, the reinterpretation and enrichment of Olympic values provide a renewed perspective on the Olympic Movement's role in addressing contemporary global challenges. The Olympic Games exert a profound influence on societies worldwide, and mass participation in sport represents a powerful strategy not only for health promotion but also for education, peacebuilding, equality, and social cohesion. Sport can further play a vital role in raising awareness of climate change and fostering environmental responsibility. National Olympic Academies and National Olympic Committees can support these goals by working in partnership with educational, sporting, and governmental institutions to promote strategic and long-term initiatives. In an era marked by heightened concern for human rights, inclusion, and sustainability, the Olympic Movement has both the opportunity and the responsibility to contribute meaningfully to education, equity, environmental stewardship, and global development and peace. A contemporary Olympic values framework can strengthen Olympic education only if translated into coherent pedagogical models, educator training, and accountable institutional practice, across schools, sport organisations, and community settings.

## Data Availability

The raw data supporting the conclusions of this article will be made available by the authors, without undue reservation.
