# Supplementary material for: Beyond excellence, friendship and respect: olympic education stakeholders’ priorities for values in a changing world
Source: Front Sports Act Living. 2026 Jun 2;8:1823613. doi: 10.3389/fspor.2026.1823613 (PMC13269054; doi:10.3389/fspor.2026.1823613)
Supplement: Supplementary file 1 [file Datasheet1.pdf]

Supplementary File

Olympic Values' Exploratory Factor Analysis

Results

Exploratory Factor Analysis (EFA) was conducted using Principal Component Analysis (PCA) with direct oblimin rotation to investigate the underlying factor structure of the Olympic Values scales. The suitability of the data for EFA was assessed using the Kaiser-Meyer-Olkin (KMO) measure of sampling adequacy and Bartlett's Test of Sphericity. Factor loading less than .30 were excluded. For each scale, two components were extracted based on eigenvalues greater than one.

Excellence

The EFA for the "Excellence" scale (N = 286) revealed a KMO value of .75, indicating good sampling adequacy. Bartlett's Test of Sphericity was statistically significant (χ^2_{15} = 651.97, p < .001), supporting the factorability of the correlation matrix. Two components were extracted, accounting for 72.24% of the total variance. More specifically, Factor 1 (affective - emotional factor) explained 51.59%, while Factor 2 (cognitive factor) explained 20.65% of the total variance. Communalities ranged from .62 to .82. The affective - emotional factor was primarily defined by Excellence6, Excellence5, and Excellence4, while the cognitive factor was defined by Excellence2, Excellence3, and Excellence1. The correlation between the two components was .36. The Pattern Matrix showed the following factor loadings:

Item	Factor 1	Factor 2
Excellence6	.849	
Excellence5	.837	
Excellence4	.828	

Excellence2	.856
Excellence3	.844
Excellence1	.766

Friendship

For the “Friendship” scale (N = 286), the KMO measure was .76, and Bartlett's Test of Sphericity was significant (χ^2_{15} = 672.05, p < .001). Two components were extracted, explaining 72.65% of the total variance. More specifically, Factor 1 (affective - emotional factor) explained 51.64%, while Factor 2 (cognitive factor) explained 21.01% of the total variance. Communalities ranged from .57 to .85. The affective - emotional factor was characterized by Friendship5, Friendship6, and Friendship4, while the cognitive factor was defined by Friendship1, Friendship2, and Friendship3. The correlation between the two components was .35. The Pattern Matrix indicated the following factor structure:

**Item**	**Factor 1**	**Factor 2**
Friendship5	.935	
Friendship6	.869	
Friendship4	.854	
Friendship1		.852
Friendship2		.817
Friendship3		.653

Respect

Analysis of the “Respect” scale (N = 286) yielded a KMO of .73 and a significant Bartlett's Test (χ^2_{15} = 699.42, p < .001). Two components were extracted, accounting

for 72.63% of the total variance. More specifically, Factor 1 (affective - emotional factor) explained 52.29%, while Factor 2 (cognitive factor) explained 20.34% of the total variance. Communalities ranged from .62 to .86. The affective - emotional factor was defined by Respect5, Respec6, and Respect4, while the cognitive factor was defined by Respect2, Respect1, and Respect3. The correlation between the two components was .43. The Pattern Matrix showed the following loadings:

**Item**	**Factor 1**	**Factor 2**
Respect5	.923	
Respec6	.902	
Respect4	.814	
Respect2		.880
Respect1		.802
Respect3		.780

Inclusion

For the “Inclusion” scale (N = 286), the KMO value was .79, and Bartlett's Test of Sphericity was significant (χ^2_{15} = 690.60, p < .001). Two components were extracted, explaining 73.95% of the total variance. More specifically, Factor 1 (affective - emotional factor) explained 54.29%, while Factor 2 (cognitive factor) explained 19.66% of the total variance. Communalities ranged from .63 to .85. The affective - emotional factor was primarily loaded by Inclusion5, Inclusion6, and Inclusion4, and the cognitive factor by Inclusion1, Inclusion2, and Inclusion3. The correlation between the two components was .46. The Pattern Matrix revealed the following factor structure:

**Item**	**Factor 1**	**Factor 2**

Inclusion5	.931	
Inclusion6	.870	
Inclusion4	.865	
Inclusion1		.856
Inclusion2		.820
Inclusion3		.796

Environment

The EFA for the “Environment” scale (N = 286) showed a KMO of .77 and a significant Bartlett's Test (χ^2_{15} = 932.47, p < .001). Two components were extracted, accounting for 77.79% of the total variance. More specifically, Factor 1 (affective - emotional factor) explained 56.89%, while Factor 2 (cognitive factor) explained 20.90% of the total variance. Communalities ranged from .70 to .90. The affective - emotional factor was defined by Environment6, Environment5, and Environment4, while the cognitive factor was defined by Environment3, Environment2, and Environment1. The correlation between the two components was .45. The Pattern Matrix indicated:

**Item**	**Factor 1**	**Factor 2**
Environment6	.969	
Environment5	.964	
Environment4	.790	
Environment3		.858
Environment2		.843
Environment1		.828

Equality

For the "Equality" scale (*N* = 286), the KMO measure was .80, and Bartlett's Test of Sphericity was significant (χ^2_{15} = 1003.81, *p* < .001). Two components were extracted, explaining 79.49% of the total variance. More specifically, Factor 1 (affective - emotional factor) explained 60.88%, while Factor 2 (cognitive factor) explained 18.61% of the total variance. Communalities ranged from .70 to .90. The affective - emotional factor was characterized by Equality6, Equality5, and Equality4, and the cognitive factor by Equality2, Equality1, and Equality3. The correlation between the two components was .53. The Pattern Matrix showed the following factor structure:

**Item**	**Factor 1**	**Factor 2**
Equality6	.972	
Equality5	.955	
Equality4	.827	
Equality2		.892
Equality1		.860
Equality3		.827

Peace

Analysis of the "Peace" scale (*N* = 286) yielded a KMO of .77 and a significant Bartlett's Test (χ^2_{15} = 980.08, *p* < .001). Two components were extracted, accounting for 78.14% of the total variance. More specifically, Factor 1 (affective - emotional factor) explained 58.69%, while Factor 2 (cognitive factor) explained 19.45% of the total variance. Communalities ranged from .63 to .91. The affective - emotional factor was defined by Peace5, Peace6, and Peace4, while the cognitive factor was defined by

Peace3, Peace2, and Peace1. The correlation between the two components was .48. The Pattern Matrix showed the following loadings:

**Item**	**Factor 1**	**Factor 2**
Peace5	.981	
Peace6	.954	
Peace4	.771	
Peace3		.900
Peace2		.897
Peace1		.705
